# Testosterone and quality of life in patients with dilated cardiomyopathy

**DOI:** 10.15190/d.2022.15

**Published:** 2022-09-29

**Authors:** Rodica Diaconu, Oana Neagoe, Oana Mirea, Eugen Tieranu, Roxana Mustafa, Tudor-Adrian Balseanu, Ionut Donoiu

**Affiliations:** ^1^Department of Cardiology, University of Medicine and Pharmacy Craiova, Romania; ^2^Department of Physiology, University of Medicine and Pharmacy of Craiova, Romania

**Keywords:** Dilated cardiomyopathy, quality of life, testosterone, Kansas City cardiomyopathy questionnaire.

## Abstract

BACKGROUND: Testosterone is an important factor that influences the quality of life in men. The purpose of this study is to evaluate how testosterone level impacts the quality of life in patients with dilated cardiomyopathy.METHODS: This cross-sectional single-center included 97 male patients with dilated cardiomyopathy, in whom serum testosterone was measured. Health-related quality of life was measured using the translated validated version of the Kansas City Cardiomyopathy Questionnaire (KCCQ-12). We used correlation and multivariable regression to assess the association between KCCQ-12 score, serum testosterone level, and clinical and paraclinical variables.RESULTS: The mean age of study participants was 58 (range 29–88). The mean LVEF was 25 ±8.61%. The average total serum testosterone level was 3.13 ±2.72 (range 0.19–13.5 ng/ml). The median global KCCQ-12 score was 44.8 (6.2–90.6) representing a poor to fair impairment in quality of life. There was an inverse correlation between the KCCQ-12 score and NYHA class (Pearson coefficient r = 0.847 p<0.001) and a direct correlation with LVEF (r=0.445, p<0.001). Also, the KCCQ-12 score correlated with hemoglobin level (r=0.214, p=0.037) and plasmatic creatinine level (r=-0.296 p= 0.004). In multivariable regression, the independent predictors of health-related quality of life were testosterone, LVEF, and NYHA class.CONCLUSIONS: The results of this study showed for the first time a significant direct relationship between serum testosterone levels and quality of life in patients with dilated cardiomyopathy.

## INTRODUCTION

Heart failure (HF) is the last stage of dilated cardiomyopathy (DCM), the most common type of disease of the heart muscle, occurring mostly in men^1^.

Patients with heart failure have a compromised quality of life. Health-related quality of life (HRQoL) has gained increasing importance in being an important outcome indicator^2^.

The Kansas City Cardiomyopathy Questionnaire (KCCQ) is the most sensitive specific and responsive health-related quality measure for heart failure being used in numerous studies since its introduction. The KCCQ-12 is the short version, which preserves the validity, reliability, responsiveness, prognostic importance, and interpretability of the original instrument. This self-administered questionnaire quantifies different assessment domains: physical limitation (range 0–100), symptom frequency (range 0–100), quality of life (range 0–100), and social limitation (range 0–100). The summary KCCQ-12 score represents the mean calculated score. A higher score is representative of a better health status^3,4^.

In men, testosterone decline with age and is one of the major factors that reduce the quality of life^5^. Low testosterone levels are frequently seen in men with chronic heart failure. Reduced testosterone levels are associated with functional capacity, depression, increased mortality, and more severe heart failure in men^6-8^.

Several studies showed that testosterone replacement therapy in patients with heart failure was associated with improved exercise capacity, cardiac function, quality of life, or clinical outcome^9,10^.

To date, no study evaluated the direct association between androgen status and HRQoL determinants in symptomatic DCM patients using the KCCQ as a health status measure. This study aimed to evaluate the correlation between serum testosterone levels and HRQoL in patients with DCM.

## MATERIALS AND METHODS

We performed a cross-sectional single-center study including 97 patients with DCM who were discharged from the Department of Cardiology of the Emergency County Hospital Craiova, Romania, between September 2020 and May 2021.

Inclusion criteria were the diagnosis of dilated cardiomyopathy: left ventricular ejection fraction <45% and/or left ventricular shortening fraction <25%, measured by transthoracic echocardiography and left ventricular end-diastolic diameter >3.2 cm/m2 (>117% of normal value) and/or left ventricular end-diastolic volume >75 ml/m2, measured by transthoracic echocardiography. Patients with significant hepatic, renal, hematologic, psychiatric, endocrine disorders, or androgen use were excluded.

The study was approved by the ethics committee of the University of Medicine and Pharmacy under registration number 43/17.06.2020. The study was conducted by the ethical principles of the Declaration of Helsinki and written informed consent was obtained before study enrolment from all patients.

In assessing the HRQoL, the patients were instructed to answer the translated validated version of the Kansas City Cardiomyopathy Questionnaire (KCCQ-12) (license from CV Outcomes Inc), independently with minimal assistance from the investigators.

Blood samples were obtained at hospital discharge each morning, with the patients in a fasted state. Blood chemistry levels: total blood count, creatinine, and glycemia, were performed at the hospital laboratory facility. In addition, blood samples from all participants were collected in tubes containing ethylenediaminetetraacetic acid, centrifugated, frozen, and transported to an associated laboratory. Serum testosterone was measured by Enzyme-Linked Fluorescent Assay. The normal range was between 2.27 to 10.3 ng/ml.

Routine echocardiographic examinations were performed blindly by experienced echocardio-graphers with standard techniques at the echo-cardiography core facility^11,12^.

### Statistical Analysis

The software programs Excel 2021 (Microsoft Corporation, Redmond, WA) and IBM SPSS Statistics 28.0 (IBM, Armonk, NY) were used for analyses. Categorical data are expressed as numbers and percentages; continuous data are presented as mean ± SD. Correlations between the KCCQ-12 score and other parameters were assessed using Person's correlation analysis. We performed multiple regression analyses, allowing for interaction between KCCQ-12 score, serum testosterone level, and other parameters. Two-sided p values ≤ 0.05 were considered statistically significant.

## RESULTS

Baseline characteristics are presented in Table 1. The mean age of study participants was 58 (range 28–88). The mean LVEF was 25 ± 8.61%. The mean eGFR was 82.95 ± 40.65 mL/min/1.73 m2. Of the study population, 36.1% (35 patients) had diabetes mellitus. The average total serum testosterone level of the study population was 3.13 ±2.72 (range 0.19 -13.5 ng/ml).

**Table 1:** Baseline Characteristics of Examined Men with DCM BMI: Body mass index; NYHA: New York Heart Association; LVEF: left ventricular ejection fraction; eGFR: estimated glomerular filtration rate.

Variables	Men with DCM (n = 97)
Age (years)	58 ± 11.9
BMI, kg/m2	27.26 ± 6.53
Diabetes mellitus, n (%)	35 (36.1)
Rhythm n (%) Sinus rhythm Atrial fibrillation	56 (57.7) 41 (42.3)
LVEF, %	25 ±8.61
NYHA class, n (%) I II III IV	4 (4.1) 26 (26.8) 32 (33) 35 (36)
eGFR, mL/min/1.73m^2^	86.97± 39.63
Hemoglobin level, g/dL	13.86±1.62
Testosterone, ng/ml	3.13 ±2.72

### Health status measured by KCCQ-12

**Figure 1:**
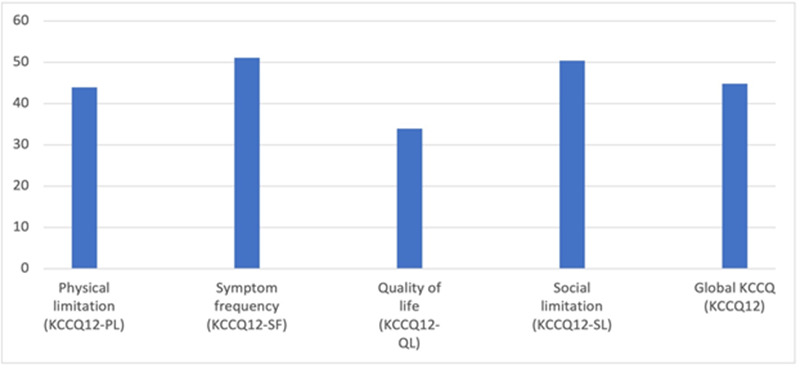
Kansas City Cardiomyopathy Questionnaire results in summary for each of the assessment domains

The global HRQoL reflected by the median KCCQ score was 44.8 (6.2–90.6) (Table 2). The symptom frequency assessment domain had the highest median score of 51.1 (0–95.8) points while the lowest median score was 33.9 (0–75) points in the quality-of-life assessment domain (Figure 1).

**Table 2:** Kansas City Cardiomyopathy Questionnaire results in summary for each of the assessment domains and global KCCQ score

Variable	Results, median (min, max)
Physical limitation (KCCQ12-PL)	43.9 (0–91.5)
Symptom frequency (KCCQ12-SF)	51.1 (0–95.8)
Quality of life (KCCQ12-QL)	33.9 (0–75)
Social limitation (KCCQ12-SL)	50.4 (0–100)
Global KCCQ (KCCQ12)	44.8(6.2–90.6)

### Correlation between KCCQ-12 score and other parameters

We correlated the clinical features and parameters of laboratory data, echocardiography, with the score of KCCQ-12 (Table 3).

**Figure 2:**
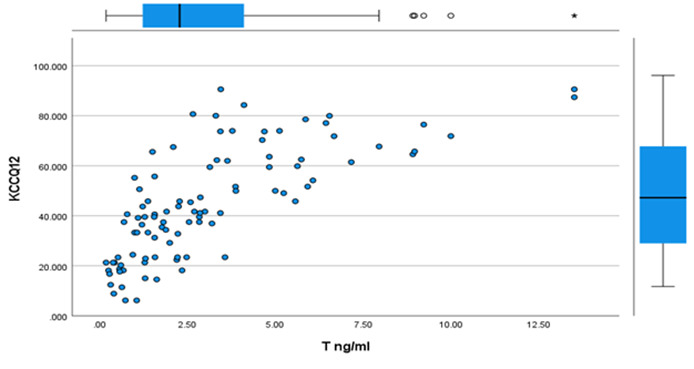
A direct relationship between KCCQ12 and Testosterone

There was a significant direct relationship between testosterone levels and quality of life (r=0.727, p <0.001) (Figure 2). The KCCQ-12 score presented strong correlations also with NYHA functional class (r=-0.847, p<0.001) and LVEF (r=0.445, p<0.001), and weak but significant correlations with hemoglobin level (r=0.214, p=0.037) and plasmatic creatinine level (r=-0.296 p=0.004). LVEF correlated especially with the symptom frequency domain (r=0.513, p<0.001).

**Table 3:** KCCQ12 score correlation analyses with serum testosterone levels and other parameters BMI: Body mass index; LVEF: left ventricular ejection fraction; NYHA: New York Heart Association.

Parameters	R	p-value
Testosterone	0.727	<0.001
Age	-0.124	0.226
BMI	-0.076	0.459
Rhythm	-0.102	0.320
Diabetes mellitus	-0.088	0.394
Serum Creatinine	-0.296	0.004
Hemoglobin level	0.214	0.037
LVEF	0.445	<0.001
NYHA class	0.847	<0.001

The strongest correlation was between testosterone and the quality-of-life domain (r=0.742, p<0.001).

### Correlation between serum testosterone and other parameters

We correlated the clinical features and parameters of laboratory data, echocardiography, with serum testosterone levels (Table 4).

**Table 4:** Serum testosterone levels correlation analyses with KCCQ score and other parameters KCCQ: Kansas City Cardiomyopathy Questionnaire; LVEF: left ventricular ejection fraction; NYHA: New York Heart Association.

Parameters	R	p-value
KCCQ	0.727	<0.001
Age	-0.224	0.027
BMI	0.049	0.633
Rhythm	-0.065	0.527
Diabetes mellitus	-0.182	0.075
Serum Creatinine	-0.247	0.016
Hemoglobin level	0.118	0.254
LVEF	0.075	0.468
NYHA class	-0.566	<0.001

Beside the direct relationship with KCCQ-12 score, serum testosterone presented strong correlations with NYHA functional class (r=-0.566, p<0.001), with plasmatic creatinine level (r=-0.247 p=0.016) and with age (r=-0.224 p=0.027).

### Multiple regression analysis of KCCQ-12 score

Regarding factors that affect the KCCQ-12 score, multiple regression analysis (Table 5) showed that testosterone, LVEF, and NYHA class are independent predictors of quality of life in patients with DCM.

**Table 5:** Multiple regression analysis (Dependent Variable: KCCQ12) T: Testosterone; Hb: hemoglobin; LVEF: left ventricular ejection fraction; NYHA: New York Heart Association

	Standardized Coefficients Beta		95% Confidence Interval	
Model	Beta	p	Lower Bound	Upper Bound
(Constant)		<0.001	20.226	69.587
T	0.443	<0.001	2.777	4.586
Age	0.025	0.588	-0.120	0.211
Diabetes	-0.023	0.594	-4.807	2.770
Hb	0.064	0.151	-.0314	2.007
LVEF	0.216	<0.001	0.308	0.804
NYHA	-0.489	<0.001	-14.876	-9.287
Creatinine	-0.073	0.113	-5.955	0.644

### Multiple regression analysis of testosterone

Regarding factors that affect testosterone level, after multiple regression analysis (Table 6) only LVEF and KCCQ-12 were independent predictors.

**Table 6:** Multiple regression analysis (Dependent Variable: Testosterone) KCCQ: Kansas City Cardiomyopathy Questionnaire; LVEF: left ventricular ejection fraction; NYHA: New York Heart Association.

	Standardized Coefficients Beta		95% Confidence Interval	
Model	Beta	p	Lower Bound	Upper Bound
(Constant)		0.393	-2.701	6.802
KCCQ12	0.989	<0.001	0.090	0.148
Age	-0.096	0.163	-0.050	0.009
Diabetes	-0.079	0.229	-1.089	0.264
Hb	-0.080	0.233	-0.336	0.083
LVEF	-0.315	<0.001	-0.142	-0.053
NYHA	0.139	0.231	-0.269	1.098
Creatinine	0.011	0.878	-0.556	0.649

## DISCUSSION AND CONCLUSION

The primary treatment goal for patients with heart failure is to alleviate their symptoms and quality of life. Patients with poor quality of life have a worse prognosis and increased severity of heart failure^1^.

KCCQ is a well-established instrument to measure the quality of life in this population. It is independently associated with mortality and hospitalizations. and U.S. Food and Drug Administration (FDA) has approved KCCQ as a clinical outcome assessment^13^.

NYHA functional classification is the most used health status measure for patients with heart failure. Like other studies, we find a strong correlation between the KCCQ-12 score and NYHA class. More and more studies support the use of KCCQ-12 in routine clinical care, showing that it could have incremental value over the NYHA classification^14,15^.

In our study hemoglobin level correlated with quality of life. Anemia is a serious co-morbidity in chronic heart failure patients. Its influence on health-related quality of life in HF patients was reported in other studies^16^. We can speculate that correction of anemia could improve life quality in these patients.

As well as anemia, chronic kidney disease is a predictor of adverse outcomes in heart failure^17^.We found a direct association between level of creatinine and life quality. Creatinine level was also correlated with testosterone level. Studies shows that low testosterone is associated with reduced kidney function. This link is multifactorial by dysregulation in the hypothalamic-pituitary-gonadal axis^18^. Kidney disease is prevalent in patients with heart failure being an independent prognostic factor^19^.

Although studies didn’t show a difference in quality of life between patients with heart failure and preserved ejection fraction and patients with heart failure and reduced ejection fraction^20^, LVEF is a powerful predictor of cardiovascular outcomes in heart failure patients. In our cohort, the LVEF was low and was associated with poor quality of life, especially in the domain of symptom frequency. Low LVEF was associated with mortality and hospitalization in patients with heart failure and in a wide range of clinical settings including during noncardiac admissions^21^.

Poor health-related quality of life is common in heart failure, but there are little data on HRQoL in HF, and how this is influenced by testosterone. Several small intervention studies had shown the links between low testosterone and impaired exercise capacity in men with HF^8-10^. The present study is the first to report an association between decreased testosterone and poor life quality in men with heart failure. Testosterone deficiency is common in men with heart failure, and studies have shown an association between low testosterone levels and poor cardiovascular outcomes^6^. In our cohort the level of testosterone was low and was associated with a lower KCCQ-12 score, especially in the quality-of-life domain, independently of older age, and cardiac function. Testosterone deficiency is associated with erectile dysfunction, decreased energy, and depression^8^, all of these being associated with life quality.

Although there are limited data on testosterone supplementation, more studies show that might be an additional therapy in men with HF, particularly in those with testosterone deficiency^6^.

As a limitation, this is a prospective cohort study of a single center with a relatively small number of patients. Also, this study included only inpatients with decompensated DCM and may not be representative of all men with HF. Further studies with a larger and more mixed population are needed.
